# The Future of the Small Friendly Societies

**Published:** 1913-02-08

**Authors:** 


### NATIONAL INSURANCE ACT DEPARTMENT.

### XLII.—The Future of the Small Friendly Societies.

Although the structure of the National Insurance Act was in the main based upon the practice and experience of the voluntary associations for thrift, known as Friendly Societies, which had existed in various forms throughout the United Kingdom for some hundreds of years, and in spite of the fact that it was the professed intention of the framers of the measure to preserve all that was best in these organisations, the result will probably be very different from what was expected.

Speaking at a meeting at the Institute of Actuaries last week, a well-informed actuary gave it as his opinion that the Act had sounded the death knell of the small Friendly Societies. If the small Friendly Societies do, in fact, disappear it will be a matter for regret from many points of view, and will tend to show that one of the most important bearings of the Act—namely, its effect on the character and habits of the people—had been seriously overlooked. It is desirable, therefore, that we should look at the matter to see how realisation has agreed with or departed from expectation. The matter is a complex one, and touches not only the financial side of the Societies, but also the social side of their operation.

Recognising the great advantages that have accrued from the Friendly Societies to those who had selected this mode of providing for sickness and incapacity, it was sought by the framers of the Act to perpetuate the system and widen its basis by requiring all employed persons, within certain limits as to income and so forth, to make like provision.

For some years the membership of Friendly Societies had shown signs of arrested growth, and the figures published by the Registrar of Friendly Societies gave evidence that voluntary thrift as expressed by membership of such Societies had its limitations. This, amongst other reasons, was one of the principal arguments used to show the necessity for augmenting voluntary effort by State aid. It was tacitly assumed that those who did not make provision for incapacity were prevented from doing so either by lack of pecuniary means or of opportunity of joining a Society. The design of the Act was to take all that was good in the Friendly Society system and confer it on those members of the industrial community who had not previously shared these advantages, whilst at the same time it was desired to encourage, as far as possible, the voluntary efforts of those who in the past had made thrift provision for themselves.

This was one of the reasons for the system of finance on which the Act is based. By that system the benefits and contributions, with minor exceptions, correspond to persons entering into insurance at the age of sixteen, and the deficiency, occasioned by this process on account of those entering insurance at higher ages, is to be made good out of the State grant until such time as it has been liquidated. As applied to individuals the system involves the crediting of reserve values to Approved Societies for those entering insurance above the age of sixteen, and no Society need be prejudicially affected by accepting such members.

### The Released Reserves.

The principal reason which now we have in mind in again drawing attention to this system of reserves is to show that a Friendly Society which has become an Approved Society for the purposes of the Act is, in certain circumstances, at an advantage as compared with newly-formed Approved Societies. The advantage lies in this, that it is possible that it may be relieved of such part of its contract with its members as is represented by the State sickness and disablement benefits, whilst the member transfers his former contribution to the private section of the Society, or such portion as is required under the Act, to the State section of the Society. The result of such an operation would be that if the Society had in the past been conducted on financially sound lines, the fund that it held to the credit of the member so transferring would be no longer required for its specific purpose. Special provisions were inserted in the Act in order to deal with these released reserves by giving increased benefits from the private funds of the Societies. In order that the matter should not be left to chance it was definitely laid down that *every* registered Friendly Society must submit a scheme dealing with the question to the Registrar.

It was thought by the framers of the Act that this advantage would be appreciated, and that it would be very generally accepted, but in the passage of the Bill through Parliament a small amendment was made in the section dealing with the matter by which the Friendly Societies might allow their members to *continue* unaltered their contributions for benefits independently of those of the Act. We now learn on reliable authority that the vast majority of the schemes submitted have followed the lines of requiring the members to continue their contributions unaltered, and even when the Societies have not adopted this course the majority of the members themselves have exercised the option of continuing to pay to their Societies as before the Act came into operation, in addition, of course, to the contributions now required to be paid to the Approved section.

It has, therefore, to be admitted that this particular section of the Act has proved a failure, and that, as so few reserves have been released, the advantages that were to accrue to the private funds of the Friendly Societies by the release of liability will not materialise to any large extent.

The Winding-up of Small Friendly Societies.

The matter is one of considerable importance, and may have far-reaching consequences, for although at first sight it may appear to be in favour of the Act that the contributions payable compulsorily thereunder are being paid in addition to former voluntary contributions, it has to be borne in mind that a large number of the Friendly Societies on their private side are financially insolvent, according to actuarial valuation. An actuarial deficiency means that in course of time, unless present conditions are altered, the Society will not be able to pay its claims as they fall due. The decay and winding-up of many a Society has only been deferred because it has been able to secure a supply of fresh young lives with which to recruit its ranks. This means of alleviation cannot be relied upon in the future in view of compulsory insurance, and it is likely that the winding-up of Friendly Societies in deficiency will be accelerated rather than retarded through the operation of the National Insurance Act. Not only will the Societies find difficulty in securing new voluntary members, but many of those who have signified their intention of continuing their contributions will, in course of time, find it impossible to fulfil their intention and will discontinue membership.

Another feature of many small Societies has been the voluntary assistance accorded by those who had in former years been active members, but, having prospered in business, have had no need to claim the sickness benefits, whilst at the same time they have been willing to support their less-favoured brethren by continuing their subscriptions. In many Societies such assistance has been on a considerable scale, and it is only natural that the State support now accorded should undermine this source of revenue. From the purely financial point of view indications are not wanting to show that the position of the Societies may be prejudicially affected by the Act. One of the principal disadvantages of the winding-up of a Friendly Society is the position in which it places its aged and infirm members. They have through a long series of years paid their contributions, and they have looked to their Society to pay the benefits in their old age. When the Society is wound up this provision is gone, and they have nowhere else to look for support. Under the National Insurance Act persons above seventy years of age do not obtain the benefits, and accordingly many old persons will necessarily suffer.

There is another reason besides the purely financial reason with which we have been dealing that may account for the winding-up of the smaller Friendly Societies. In the past the Secretary has been willing to do his work voluntarily, or for a nominal consideration, but under the arrangements now existing it is impossible for this to continue. The duties that now devolve upon the Secretaries of those Societies that have become Approved are such as to require the services of experts. This is exemplified by the Handbook which has been recently published by the Insurance Commissioners for the guidance and assistance of Secretaries of the smaller Societies in the administration of sickness and maternity benefits. This publication is in all respects most admirable, but a casual reference to it will immediately show the complex nature of the duties to be performed and the impossibility of their being adequately carried out except by persons specially trained to the task.

Thus the very strength of the Friendly Societies in the past may prove to be their source of weakness in the present, and it would seem that the future of the small Friendly Societies is anything but desirable. Already we understand that large numbers of these smaller local Societies have taken the necessary steps for dissolution. The country will be poorer by their loss, because they have not only served a useful purpose in dispensing relief in sickness, but by mutual intercourse of the members they have stimulated the spirit of self-help as expressed in unselfish and voluntary corporate action, and any loss of such qualities must necessarily be to the disadvantage of the community in general.

**NOTE.—Questions and Correspondence on all doubtful points are invited, and should be addressed to the Editor, The Hospital, 28, 29 Southampton Street, Strand, London, W.C., and marked "Insurance."**

